# Effects of combined Mediterranean diet and physical activity intervention on the gut microbiome and disease progression in individuals with Parkinson’s disease: study protocol for a multicenter, randomized controlled pilot study (PRIME study)

**DOI:** 10.3389/fnagi.2026.1743490

**Published:** 2026-03-25

**Authors:** Alice Fognani, Rossella Rotondo, Maria Luisa Savo Sardaro, Antonio de Iure, Maria Gaglione, Miriam Casali, Lorenza Leonardi, Fabiana Giada Radicati, Laura Vacca, Michele Guescini, Sabrina Donati Zeppa, Lucia Gatta, Fabrizio Stocchi, Barbara Picconi, Maria Francesca De Pandis

**Affiliations:** 1 Department for the Promotion of Human Science and Quality of Life, San Raffaele University, Rome, Italy; 2 IRCCS San Raffaele Roma, Rome, Italy; 3 IRCCS San Raffaele Roma, Cassino, Italy; 4 Department of Anthropology, Northwestern University, Evanston, IL, United States; 5 Department of Biomolecular Sciences, University of Urbino Carlo Bo, Urbino, Italy; 6 Scientific Direction, IRCCS San Raffaele Roma, Rome, Italy

**Keywords:** Mediterranean diet, microbiome, Parkinson’s disease, PD biomarkers, physical activity, pilot study, study protocol

## Abstract

**Background and aims:**

Parkinson’s disease (PD) is a neurodegenerative disorder for which no disease-modifying therapy currently exists, making it crucial to investigate alternative strategies that may slow its progression. The PRIME study will investigate the effects of combined dietary and physical activity interventions- performed in rehabilitative settings with health professional supervision and evaluation, versus single interventions on the gut microbiome in PD. The aim is to identify microbiome profiles – comparing traditional 16s rRNA gene sequencing with the third-generation method – as potential non-invasive, stage specific biomarkers of PD. In addition, the study will assess whether the combined intervention affects disease progression, symptoms, cognitive abilities, and quality of life.

**Methods:**

Eighty participants with PD will be randomized into four arms: a Mediterranean-diet intervention group; a structured physical-activity group; a combination group receiving both dietary and exercise interventions; a control group receiving standard care only.

**Conclusion:**

By integrating microbiome characterization with the evaluation of these interventions, the study aims to explore whether intervention-induced changes in the microbiota are associated with clinical improvement in PD, thus paving the way for the design of future non-pharmacological protocols to slow disease progression, mitigate symptom severity, and promote diagnosis.

**Clinical trial registration:**

https://clinicaltrials.gov/, identifier NCT07097103.

## 1 Introduction

According to the revised definition proposed by Berg et al. (2020), microbiota denotes the complex community of microorganisms inhabiting a given environment (for example, the human gut), including representatives from multiple kingdoms (Bacteria, Archaea, Fungi, Protists, and viruses). By contrast, the microbiome comprises the microbial community together with its “theater of activity” — that is, microbial structural elements, the collective genomes, metabolites and signaling molecules, and the local environmental context that mediate microbe–host interactions. Recent advances in high-throughput sequencing technologies and the development of robust, re-producible bioinformatic workflows have substantially improved taxonomic and functional resolution in microbiome studies and increased the accessibility of large-scale analyses (Knight et al., 2018). Accumulating evidence indicates that the gut microbiome (GM), which is the most extensively studied human microbial community (Cryan et al., 2019), plays a central role in host physiology (Ross et al., 2024; Cryan et al., 2020). Perturbations of the gut microbial ecosystem - known as dysbiosis - have been associated with impaired homeostasis and with a wide range of pathological processes. Mechanistically, the GM may influence the host through multiple complementary pathways that exert immunomodulatory and neuromodulatory effects: production of short-chain fatty acids (SCFAs; e.g., butyrate, propionate), modulation of bile acid pools, generation of tryptophan-derived metabolites, release of microbe-associated molecular patterns such as lipopolysaccharide (LPS), alteration of intestinal barrier integrity, and signaling along the vagus nerve. These mechanisms provide biological plausibility for microbiome–immune–brain interactions, but the relative contribution of each pathway remains to be fully delineated (Cryan et al., 2019).

Bidirectional interaction between the GM and brain-resident immune cells (microglia) have received considerable attention as potential mechanism linking the gut and the brain (Cao et al., 2025). Microglia are the central nervous system’s (CNS) resident innate immune cells; they are highly ramified and plastic and, once activated, can release cytokines and chemokines, express antigen-presenting molecules, modulate neurotransmitter systems and undergo marked morphological and functional changes (Cardinale et al., 2021). Experimental studies in germ-free animals demonstrate that a diverse GM is necessary for normal microglial development and maturation, and that recolonization or administration of microbial metabolites can partially restore microglial phenotype and function (Cryan et al., 2019; Erny et al., 2015). These preclinical findings support the hypothesis that a healthy and diverse GM contributes to the maintenance of microglial homeostasis and, by extension, to normal brain function (Cryan et al., 2019); however, translational extrapolation to humans should be made cautiously. Neurological disorder - a group of conditions affecting the nervous system including CNS and enteric nervous system (ENS) - are characterized by a rage of motor and non-motor symptoms that may reflect interrelated processes of neuroinflammation and neurodegeneration. These mechanisms are tightly interconnected in the development and progression of many neurological disorders, including Parkinson’s disease (PD) (Stolzer et al., 2023). At present, PD is considered a multifactorial disorder with an overall progressive course and no established disease-modifying therapy (Stolzer et al., 2023). Because the GM can influence systemic inflammatory status (Wastyk et al., 2021), interventions that modulate microbiome composition and function may hold therapeutic or preventive potential. Importantly, whereas the human genome is essentially stable throughout life, the microbiome is highly dynamic and responsive to external inputs, rendering it an attractive target for modification (Cryan et al., 2019). Fecal microbiota transplantation from PD patients has been reported to exacerbate disease-related phenotypes in animal models, indicating the presence of specific disease-promoting microbes (Sampson et al., 2016). Moreover, Li et al. (2017) observed PD-associated shifts in the GM — progressive loss of fiber-degraders and gain of putative pathobionts — likely resulting in reduced SCFA levels and increased endotoxin and neuroactive metabolite production. Multiple factors shape the GM across the lifespan, including host genetics, immune interactions, medication use (notably antibiotics), early-life exposures, environmental and geographic influences, sleep and mental health; among these determinants, diet and physical activity are particularly amenable to intervention (Gilbert et al., 2018). A central question motivating this study is whether lifestyle intervention can modify gut micro-biome composition and the systemic inflammatory profile—thereby altering disease progression—and whether microbial signatures that vary with disease stage may serve as early-stage biomarkers.

Despite increasing interest in lifestyle-based strategies to modulate the GM in PD, intervention evidence remains limited and heterogeneous. Studies have evaluated dietary patterns or physical activity separately, but they vary in intervention delivery, intensity, and outcome selection; to our knowledge, this is the first randomized study to evaluate a combined diet-plus-exercise program while concurrently assessing the GM and progression-related clinical outcomes. Moreover, most microbiome studies rely on short-read 16S rRNA sequencing, which may limit taxonomic resolution for biomarker exploration, and longitudinal data beyond end-of-intervention are scarce. PRIME was designed to address these gaps through a multicenter randomized factorial pilot trial comparing Mediterranean diet, intensive physical activity, and their combination versus standard care, with a prespecified primary microbiome endpoint and exploratory follow-up assessments.

## 2 Study objectives

The Parkinson’s Research In Metagenomic Early-stage biomarkers (PRIME) study aims to evaluate the impact of a combined dietary and physical activity intervention, performed in rehabilitative settings with health professional supervision and evaluation, compared with each single intervention (diet and physical activity alone), on the GM of patients with PD, with the ultimate goal of identifying a specific microbiome profile that could serve as an non-invasive, stage-specific biomarkers. The study will also explore whether the combined intervention modifies are associated with the disease progression, as measured by changes in motor and non-motor symptoms (including gastrointestinal manifestations), cognitive performance, and health-related quality of life.

## 3 Materials and methods

### 3.1 Study design and setting

**FIGURE 1:**
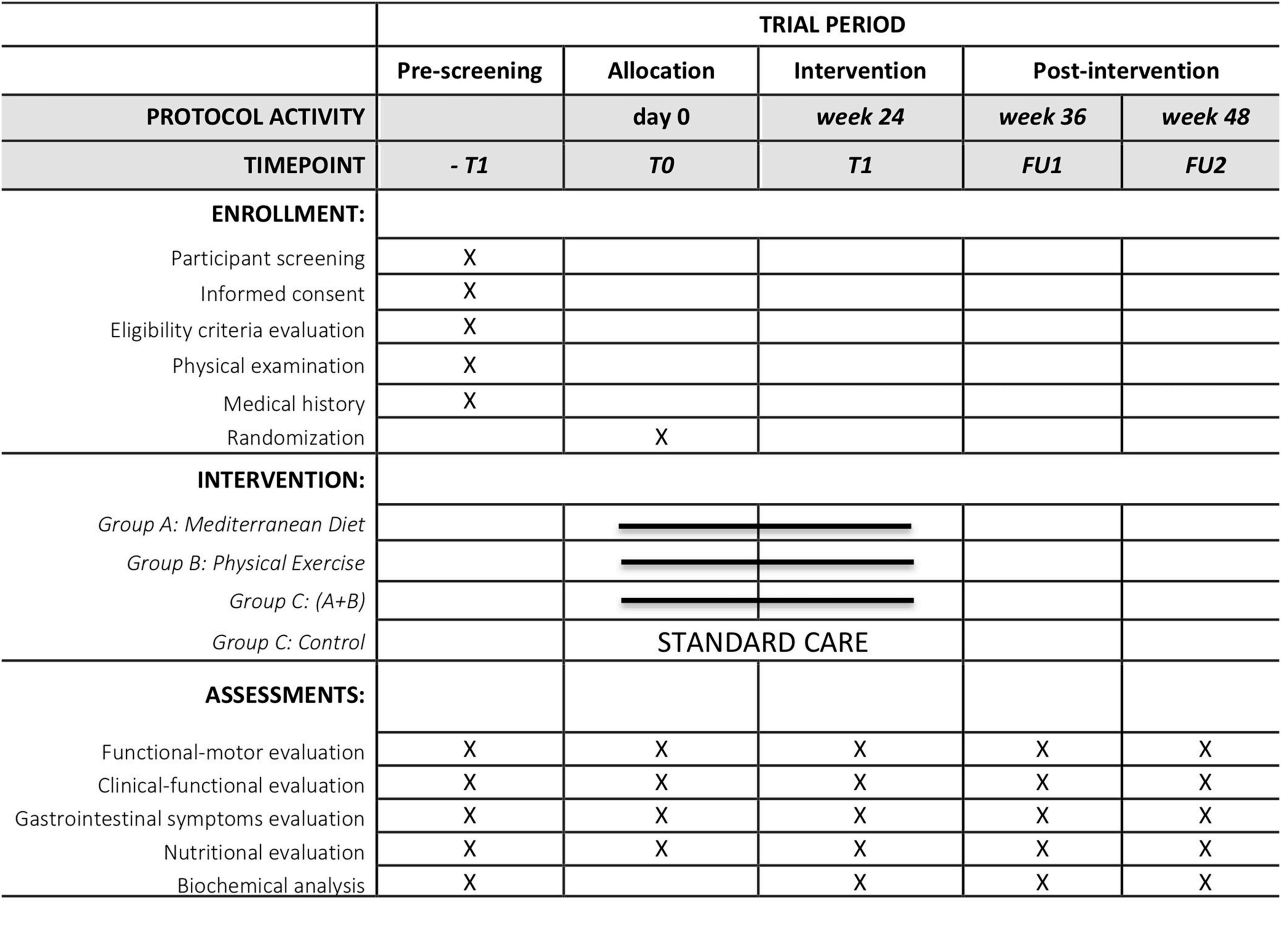
Schedule of enrollment, interventions, and assessments (SPIRIT figure). X indicates that the corresponding assessment and/or biological sample collection will be performed at that time point.

The PRIME study is a 6-month, parallel-group, four arm, open-label pilot trial employing a 2 × 2 factorial, randomized, multicenter, controlled design conducted in accordance with the SPIRIT guidelines (Chan et al., 2025; Figure 1 and Supplementary Table 1).

This multicenter study will be conducted at two Parkinson’s Disease Centers located at IRCCS San Raffaele Roma (RU1), which will serve as the Coordinating Center, and San Raffaele Cassino (RU2), as a participating clinical site. The study will be carried out in close collaboration with academic centers of San Raffaele University of Rome (RU3); Northwestern University (Evanston, Chicago, IL, United States) (RU4); University of Urbino Carlo Bo (RU5), that will act as partner research sites.

The study protocol was reviewed and approved by the Ethics Committee of Lazio Area 5 (RP 340/SR/25; Protocol version 1.0; number: 0009232, date 19 June 2025). The trial will be conducted in accordance with the Declaration of Helsinki and with applicable data protection laws, including the General Data Protection Regulation (EU) 2016/679 (GDPR). Before enrolling in the trial, each participant will be comprehensively informed about the study’s objectives, procedures, potential risks, and benefits. Adequate time will be provided for individuals to ask questions, receive clarifications, and consider their decision. Participation will be entirely voluntary, and only upon the signature of a study-specific consent form, will the individual be included in the trial.

This protocol was drafted solely on the basis of clinical and scientific expertise without direct patients or public involvement, although we recognize the importance of such engagement in research.

The trial will follow CONSORT guidelines (Schulz et al., 2010) and has been registered on ClinicalTrials.gov (NCT07097103; registered 30 July 2025) in accordance with the International Committee of Medical Journal Editors (ICMJE) recommendations. Supplementary Figure 1 shows the planned participant flow diagram (Hopewell et al., 2025) in line with CONSORT recommendations that illustrated the expected enrollment, allocation, follow-up and analysis stages for the trial. The full completed diagram will be presented with the final trial results.

### 3.2 Participants and eligibility criteria

The calculation of the sample size was not strictly required given the pilot nature of this study. Accordingly, the target sample size was primarily driven by feasibility considerations and the need to obtain precise effect-size estimates to inform a definitive RCT. However, to support the microbiome endpoint, we performed an *a priori* power assessment. Following the recommendations of Ferdous et al. (2022), we selected β-diversity as the primary endpoint to detect differences between groups in the composition of intestinal microbial species. Using G*Power version 3.1.9.7, assuming an effect size of (Cohen’s f) 0.25, a statistical power of 0.95 (1−β) and setting α to 0.05, we estimated the minimum required sample size. To account for expected attrition during the intervention and follow-up period (approximately 10%), the total sample size was increased to 80 participants (20 per arms). Participants will be recruited from Clinical Trial Centers for Parkinson’s Disease (RU1 - RU2) during routine clinical visits. A total of eighty (*n* = 80) participants meeting the following eligible criteria will be enrolled and randomly assigned to an intervention or to the control group:

#### 3.2.1 Inclusion criteria

- Males and females aged 35–80 with a diagnosis of Parkinson’s disease according to the United Kingdom (UK) Parkinson’s Disease Society Brain Bank criteria (Gibb and Lees, 1988);
- Disease stage I-III in the “ON” phase, as defined by the modified Hoehn and Yahr (H&Y) scale (Hoehn and Yahr, 1967);
- No severe cognitive impairment according to the Mini-Mental State Examination score (MMSE ≥ 24) (Folstein et al., 1975) and the Montreal Cognitive Assessment score (MoCA ≥ 17.54/30) (Santangelo et al., 2015);
- Stable dopaminergic pharmacological treatment (for at least 4 weeks prior to screening);
- Suitable for physical exercise and able to walk unassisted (no walking aids);
- Willingness to maintain usual diet during a ≥4-week run-in period;
- Willingness to adopt a Mediterranean style diet during the intervention;
- Ability to provide stool samples at each collection timepoint;
- Willingness to abstain from strenuous exercise and alcohol for 24 h before each study visit;
- Ability to interact with the research team and provide informed consent in Italian language;
- Willingness and ability to comply with all study procedures.

#### 3.2.2 Exclusion criteria

- Atypical or secondary Parkinsonism;
- Pre-existing psychiatric disorders or dementia diagnosis;
- Implanted subcutaneous electronic device (e.g., pacemaker);
- History of deep brain stimulation (DBS) surgery;
- Moderate to severe cognitive impairment;
- Beck Depression Inventory II (BDI II) score ≥ 28;
- Active serious illness (e.g., thyroid dysfunction, type 2 diabetes with HbA_1_c ≥ 8% or on insulin, active malignancy, type 1 diabetes) or unstable condition requiring medical dietary supervision (e.g., IBD, IBS, celiac disease);
- Acute medical illness (including antibiotic-resistant infections);
- Major gastrointestinal surgery or acute gastrointestinal conditions (e.g., gastroenteritis) within the past 3 months;
- Chronic corticosteroid therapy;
- Use of proton pump inhibitors within the past 30 days;
- Antibiotic intake within the past 30 days;
- Prebiotic and/or probiotic use within the past 30 days;
- Prolonged use (≥3 months) of anxiolytic drugs, antidepressants, antipsychotics, cognitive stimulants, or analogues;
- Underweight: body mass index (BMI) < 18.5 kg/m^2^;
- Pregnancy or lactation;
- Regular use of enemas or suppositories for constipation;
- Participation in other experimental protocols within 3 months of screening;
- Requirement for special dietary regimens incompatible with Mediterranean diet (e.g., strict vegan or vegetarian patterns).

### 3.3 Dropout criteria

Participants may withdraw from the study at any time for any of the following reasons: (i) desire to leave the study (ii) non-compliance or refusal to cooperate with study procedures or investigators (iii) a medical condition that, in the investigator’s judgment, contraindicates further participation. In each case, the reason for withdrawal will be documented in the study records.

### 3.4 Procedures

At screening visit, demographic data, disease duration and detailed medical history including medications will be collected. Then, all participants will undergo comprehensive neurological and neuropsychological evaluations, including the administration of standardized neuropsychological tests and scales, such as MoCA, MMSE, and BDI-II. The disease stage will be assessed using modified H&Y scale, while participants will also complete a detailed 3-days dietary record for nutritional assessment (see Supplementary Table 1).

#### 3.4.1 Randomization, timeline and masking

Enrolled participants will be sequentially randomized upon receipt of informed consent and availability of medical records, using a computer-generated, permuted-block scheme (1:1:1:1) prepared in advance by an independent statistician. Allocation will be stratified by disease stage and age group to ensure balance across arms. Each participant will be informed of their assignment at the baseline visit (T0). Moreover, given the nature of the dietary and physical-activity interventions, this is an open-label trial and participants and intervention providers cannot be blinded. To mitigate performance and assessment bias, all clinical assessments will be performed according to standardized operating procedures and by trained assessors, following a prespecified assessment order. Motor assessments will be performed in the ON state and scheduled at a consistent time relative to the participant’s usual dopaminergic medication intake across visits. Patient-reported questionnaires will be administered using standardized instructions and the same mode of administration across visits. Laboratory and bioinformatics analyses will be performed on coded samples, with analysts blinded to group allocation and timepoint. To ensure data integrity, a researcher non-involved in clinical evaluation or in the collection and management of biological samples, will enter the information into the database.

**FIGURE 2:**
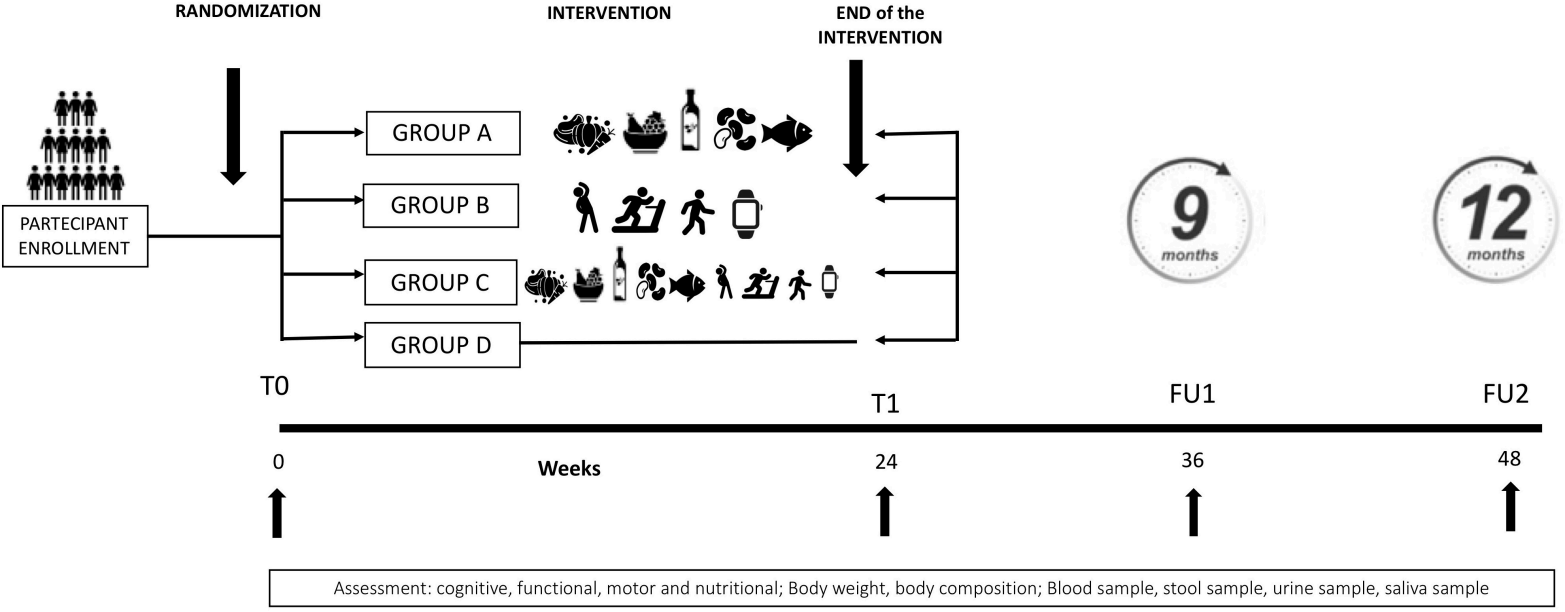
Organization of the intervention study. T0, baseline (week 0); T1, end of intervention (week 24); FU1, first follow-up (week 36); FU2, second follow-up (week 48).

The study timeline (Figure 2) comprises four clinical assessments and concomitant biological sample collections:

- T0 (baseline): pre-intervention assessment (Week 0)
- T1 (end of intervention): 6 months after the start of the intervention (Week 24)
- FU1 (first follow-up): 3 months post-intervention (Week 36)
- FU2 (second follow-up): 6 months post-intervention (Week 48).

This longitudinal design allows sufficient time to characterize temporal changes in GM composition and diversity (Clauss et al., 2021; Pagliai et al., 2020).

#### 3.4.2 Anthropometric and body composition

A registered nutritionist will measure each participant’s height and weight to calculate BMI, and will assess body composition using a Bioelectrical Impedance Analyzer device (TANITA).

#### 3.4.3 Physical exercise supervision

Physical exercise sessions will be supervised by health professionals and Adapted Physical Activity Specialists to ensure safety, proper technique, and adherence to the training protocol, as well as to guarantee accurate quantification of daily exercise intensity and weekly exercise volume. Physical activity will also be monitored using a smartwatch to record and quantify individual exercise parameters. To further ensure participant safety, falls will be prospectively recorded at each visit and through falls diary completed by participant, and musculoskeletal adverse events (e.g., muscle strain, joint pain, or injury) will be systematically assessed.

#### 3.4.4 Collection and storage of biological samples

Biological samples will be collected at each outpatient visit and all samples will be processed according to standard operating procedures and stored under optimal conditions. Specifically, 10 mL of blood will be collected in ethylenediaminetetraacetic acid (EDTA) tubes for Ficoll-Paque density gradient plasma separation and peripheral blood mononuclear cells (PBMCs) isolation; an additional 6 mL in EDTA tubes for plasma multiplex immunoassay (Luminex) and 5 mL in serum-separator tubes for future research analysis will be collected (Becton-Dickinson).

For the fecal samples, participants will be instructed to collect approximately 10 g of feces into two pre-labeled tubes, one of which contains an appropriate preservative solution. One tubes will be used for microbiome analysis, the other for further research about SCFA analysis. During each visit, a total of 4 ml of saliva will be collected in two cryovials for Extracellular Vesicles (EVs) and oral microbiota analysis. In addition, a urine sample will be collected for further biochemical and microbiological assays. Each specimen will be labeled with a unique participant code, recorded in a secure biorepository database, and stored in accordance with best clinical practices. The samples will be kept for the duration of the project, exclusively for research purposes; any use or disposal will be properly documented.

#### 3.4.5 Intervention groups

Eligible participants will be randomized to one of four six-month arms (Figure 2):

- Group A: Mediterranean diet

Participants will receive a detailed and personalized 7-days menu (specifying food ingredients by weight and/or volume), developed by a nutritionist considering: (1) PD pharmacological treatment timing and absorption interaction, (2) core Mediterranean diet principles and (3) typical Italian meal patterns (e.g., five daily eating occasion). No food category will be excluded *a priori*; however, red and processed meats will be limited to once per week, poultry to ≤3 servings/week and fish to 2/3 servings/week. Substitutions will be provided. Macronutrient targets are approximately 25%–30% of energy from fats, 15%–20% from proteins and 55%–60% from carbohydrate (primarily complex). In particular, extra-virgin olive oil will be recommended as the main added fat; daily intake of plant foods (fruit/vegetables and legumes) and preference for whole-grain products will be emphasized; and added sugars and ultra-processed foods will be discouraged. No meals or supplements will be provided. Participants will prepare their own meals or eat at a restaurant. Dietary adherence will be assessed using a validated Mediterranean diet adherence questionnaire (the MediLITE) (Sofi et al., 2017), administered at each timepoint. This will be complemented by structured dietary records (e.g., 3-day food diaries - including one weekend day) collected periodically to quantify key components; participants will receive prior standardized training to ensure accurate and consistent completion of the records.

- Group B: intensive physical activity

According to the World Health Organization [WHO] (2020) guidelines, there is a dose–response relationship between physical activity and health outcomes, with greater health benefits achieved with increasing amounts of physical activity, and the largest relative gains observed when moving from inactivity to moderate activity levels. Consistently, our ongoing study (Rotondo et al., 2024) suggests a similar dose–response effect in individuals with PD, where higher volumes of structured exercise (expressed in MET-minutes per week) are associated with improvements in both functional outcomes and peripheral biomarkers. In this regards, participants to PRIME study will follow World Health Organization [WHO] (2020) recommendations for adults, including those aged ≥ 64 years: 150–300 min/week of moderate-intensity aerobic physical activity; or 75–150 min/week of vigorous-intensity aerobic physical activity, corresponding to approximately 500–1,000 MET-min per week (moderate intensity: 3.0–5.9 METs; vigorous intensity: ≥6.0 METs); or an equivalent combination; plus muscle-strengthening exercises of moderate or higher intensity for all major muscle groups on ≥2 days/week. The daily intensity and weekly volume of physical exercise will be supervised by Adapted Physical Activity Specialists and health professionals, and monitored via smartwatch.

- Group C (A + B): Mediterranean diet + intensive physical activity combined intervention

Participants will concurrently adhere to the dietary and physical-activity protocols described above.

- Group D: controls

Participants will continue usual PD management according to clinical guidelines, without any additional specific dietary or exercise interventions provided from the study. To quantify potential contamination, diet adherence and physical activity levels will also be monitored in the control arm using the same dietary adherence tools (MediLITE and structured dietary records) and standardized physical-activity questionnaires/brief activity logs.

To optimize adherence to the program, reminders will be sent via phone calls, email or text message before each study visit. All participants will be followed up by physicians and nutritionists with weekly or monthly phone or videocalls or outpatient periodically visits to reinforce compliance, improve adherence to diet and/or physical activity, and monitor for adverse events. Physical exercise will be supervised and monitored by Adapted Physical Activity Specialists and health professionals to ensure safety, correct execution, and appropriate training progression.

### 3.5 Study outcomes

#### 3.5.1 Primary outcome

The primary endpoint of the PRIME study is to evaluate the effect of the interventions on GM β-diversity in PD patients, defined as the change in Bray-Curtis dissimilarity from T0 (baseline) to T1 (end of intervention). The prespecified primary contrast will be the between-group difference in the T0-T1 change in β-diversity for the combined intervention (Group C) versus standard care (Group D). As supportive primary microbiome descriptors, we will also quantify α-diversity and taxonomic composition/relative abundance, with analyses primarily intended to describe community structure and to support interpretation of β-diversity findings. Microbiome profiling will be performed using third-generation, high-resolution long-read sequencing technology, the Pacific Biosciences (PacBio) system, capable of sequencing amplicons up to ∼20,000 base pairs (bp) (Biada et al., 2025; Buetas et al., 2024). Exploratory microbiome aims will include (i) identification of stage-associated microbial patterns and candidate non-invasive microbiome biomarkers, treated as hypothesis-generating, and (ii) a methodological comparison between PacBio long-read sequencing and Illumina MiSeq short-read 16S rRNA amplicon sequencing. The comparison will evaluate relative performance in terms of taxonomic resolution and community profiling, with particular attention to the ability of PacBio to achieve higher resolution at the species level. We will also assess the degree of compositional similarity between microbial communities characterized by the two platforms, thereby determining the feasibility and potential benefits of integrating sequences from different technologies in microbiome research. FU1 and FU2 assessments will be used for exploratory analyses of the durability/maintenance of microbiome changes beyond the end of the intervention. Results from these exploratory analyses will be interpreted in the context of the pilot design.

#### 3.5.2 Secondary outcomes

Secondary outcomes will include biochemical, clinical, cognitive and gastrointestinal measures, to provide a broader assessment of the intervention’s effects beyond GM composition. All secondary clinical and biomarker outcomes will be considered exploratory in this pilot trial and will primarily provide feasibility metrics and effect-size estimates to inform a definitive RCT. In details:

1.
  a.    Biochemical analyses will include:
2. Changes in zonulin (biomarker of intestinal permeability) assessed by measurement of fecal zonulin concentration (reported in ng/mL) using an ELISA kit.
3. Changes in calprotectin (biomarker of intestinal inflammation) assessed by quantitative measurement of fecal calprotectin (reported in μg/g feces) using a validated immunoassay (ELISA or automated chemiluminescent assay) on stool sample.
4. Assessment of EVs size (nanometers) and concentration (particles/mL) in plasma and saliva using nanoparticle tracking analysis (NTA) and transmission electron microscopy (TEM).
5. Phenotyping of EVs in plasma and saliva through the analysis of EV-associated proteins, using fluorescent NTA (f-NTA), ELISA, and flow cytometry.
  a.    Functional-motor assessments will include:
  b.    Changes in Movement Disorder Society—Unified Parkinson’s Disease Rating Scale (MDS-UPDRS) parts II (motor aspects of daily living), III (motor examination), and IV (motor complications), a widely validated measure of PD progression (Goetz et al., 2007).
  c.    Changes in walking speed and gait-related spatiotemporal parameters by using a wearable inertial sensor device (G-sensor, BTS Bioengineering, Milan) (Zago et al., 2018), including:
  d.    Stride length (m), defined as the distance between two successive heel strikes of the same foot;
  e.    Stride length/height (%), the stride length normalized by subject height;
  f.    Cadence (steps/min), the number of steps taken per minute;
  g.    Propulsion (m/s^2^), the anterior–posterior acceleration during the swing phase of the lower limb;
6. Changes in execution timing of Time Up and Go, a validated measure of mobility, balance, walking ability, and fall risk (Kleiner et al., 2018).
7. Changes in Berg Balance Scale (BBS), a standardized tool for evaluating static and dynamic balance (Qutubuddin et al., 2005).
8. Clinical-functional assessments will include:
9. Changes in cognitive function through the administration of MMSE, MoCA, Frontal Assessment Battery (FAB), and clock-drawing tests, which are widely used to assess multiple cognitive domains (Folstein et al., 1975; Nasreddine et al., 2005; Bezdicek et al., 2017).
10. Changes in depressive symptoms, measured with the Beck Depression Inventory-II (BDI-II), a 21-item self-report scale for depression severity (Steer et al., 1998).
11. Changes in non-motor symptoms, assessed with MDS-UPDRS part I (non-motor daily living experiences) and the Non-Motor Symptoms Scale (NMSS) in PD (Van Wamelen et al., 2021).
12. Changes in quality of life, measured with the Parkinson’s Disease Questionnaire (PDQ-39), which evaluates eight domains of daily functioning (Jenkinson et al., 1997).
13. Gastrointestinal assessment will include:
14. Changes in gastrointestinal function, measured through the administration of Gastrointestinal Symptom Rating Scale (GSRS) (Rusch et al., 2024; Kenna et al., 2021), the Bristol Stool Form Scale (Lewis and Heaton, 1997; O’Donnell et al., 1990), and the Digestion-Associated Quality of Life Questionnaire (DQLQ) (Beke et al., 2022).

### 3.6 Adverse events

Although the interventions are non-pharmacological and are expected to pose minimal risk, adverse events that may occur will be those normally associated with standard clinical treatment for PD. For this protocol, an adverse event is defined as any unfavorable or unintended sign, symptom, abnormal laboratory result, or medical condition that arises during study participation, regardless of causality. Adverse events will be actively monitored throughout the intervention and follow-up, with specific attention to events potentially related to physical activity (e.g., falls and exercise intolerance) and to gastrointestinal symptoms (e.g., constipation and abdominal pain) potentially related to dietary changes. Participants will receive standardized instructions on safe exercise execution and on when/how to contact the study team. Exercise sessions will be supervised. All such events will be documented and reviewed by the principal investigator and the research team to assess severity and any link to study participation, with appropriate clinical management and follow-up until resolution or clinical stability.

### 3.7 Data collection and management

All data will be systematically recorded in Research Electronic Data Capture (REDCap), a secure web-based application for building and managing online surveys and databases. Participants will be identified only by a unique study identification number (ID) to ensure anonymity; no personally identifiable information will be stored in the electronic database. Hard-copy linkages between participant IDs and contact details will be retained in a locked file cabinet within a secure office, with access restricted to designated members of the research team. Participant files and other source data—including questionnaires, original test reports, informed consent records, and all other study-related documents—will be retained for the maximum period permitted by institutional policy.

## 4 Statistical analysis

Statistical analyses will be conducted using IBM Statistical Packages for Social Science Software (SPSS) version 23 (IBM Corp., Armonk, NY, United States) and R (R Foundation for Statistical Computing, Vienna, Austria) for microbiome-specific analyses. A *p*-value < 0.05 will be considered statistically significant for the prespecified primary analysis; multiplicity control will be applied where appropriate for exploratory high-dimensional microbiome analyses using false discovery rate procedures (e.g., Benjamini–Hochberg). Baseline characteristics will be summarized using descriptive statistics [mean ± standard deviation (SD) for normally distributed data, or as median with interquartile ranges (IQR) in case of asymmetric distributions]. Categorical variables will be expressed in terms of absolute frequencies and percentages. Comparisons across the four study groups will be conducted using statistical tests appropriate to the data type and distribution for outcomes assesses at a single time point (ANOVA or Kruskal-Wallis for continuous variables; Chi-square/Fisher’s for categorical variables). Given the longitudinal design (T0, T1, FU1, FU2), outcomes measured repeatedly over time will be analyzed using mixed-effects models to account for within-participant correlation. Analyses will be conducted primarily under an intention-to-treat principle; sensitivity analyses will assess the impact of missing data where relevant. For continuous outcomes, linear mixed-effects models will include fixed effects for time, diet, exercise, and their interactions (time × diet × exercise) (or time × trial arm), and a random intercept for participant. For non-normally distributed outcomes, appropriate transformations or generalized mixed models will be considered. The prespecified primary contrast will focus on the change from T0 to T1 in the combined intervention versus standard care (control); analyses including FU1 and FU2 will be considered secondary/exploratory and reported as durability/maintenance assessments. In addition, subgroup analyses will be conducted to explore potential heterogeneity of intervention effects by disease stage (I/II/III of H&Y), age group, and gender, where sample size permits, with results interpreted as exploratory. Sensitivity analyses will be conducted to assess the robustness of findings, including adjustment for prespecified covariates and assessment of protocol adherence where relevant.

### 4.1 Fecal microbiome processing, sequencing, bioinformatics, and statistical analysis

Fecal collection kits and detailed instructions will be provided to participants. Samples will be stored at −80 °C until processing. DNA will be extracted using the PowerSoil Pro DNA kit (Qiagen). For short-read profiling, the V4–V5 region of the 16S rRNA gene will be amplified using primers 515F and 926R with Illumina barcodes. For long-read profiling, the 16S-ITS-23S operon will be amplified with primers A515F and U2428R and PacBio barcodes to obtain amplicons > 3,500 bp. Sequencing of both short- and long-read libraries will be performed by the Sequencing Core at the University of Illinois at Urbana-Champaign (UIUC). Raw sequence data will be processed in the R statistical environment; DADA2, a bioinformatics pipeline, specifically developed for modeling and correcting amplicon sequencing errors (Callahan et al., 2016), will be used for quality assessment, length filtering, denoising high-quality reads into Amplicon Sequence Variants (ASVs) and chimera removal.

Given the complexity of the dataset, microbiome statistical analysis will be conducted using standardized and reproducible methodologies. α- and β-diversity analyses will be assessed at appropriate taxonomic levels, from phylum to genus, using the phyloseq package in R (McMurdie and Holmes, 2013). α-diversity will be estimated using indices such as Shannon, Simpson, and Faith’s phylogenetic diversity and between-group differences will be tested using appropriate non-parametric test (e.g., Kruskal-Wallis) for cross-sectional comparisons or generalized linear models adjusted for covariates. For longitudinal analyses across T0, T1, FU1, and FU2, mixed-effects models will be used where appropriate to account for repeated measures within participants. β-diversity will be quantified using distance metrics including Bray-Curtis, Jaccard, and weighted/unweighted Unifrac. The prespecified primary microbiome analysis will focus on Bray–Curtis β-diversity and will evaluate whether the change from T0 to T1 differs between the combined intervention (Group C) and standard care (Group D) using Permutational Multivariate Analysis of Variance (PERMANOVA). To account for the repeated-measures structure, permutation testing will be restricted within participants. FU1 and FU2 will be included in secondary/exploratory analyses of durability/maintenance.

To evaluate the effect of sequencing platform and interindividual variability, (PERMANOVA) will be applied. Concordance between PacBio and Illumina community profiles will be assessed using Mantel and Procrustes tests and reported as exploratory. Taxonomic composition will be analyzed using complementary approaches such as ANCOM-BC (as primary compositional method) and DESeq2, LEfSe as exploratory approaches, to identify taxa or pathotypes showing significant differences between experimental groups. Multiple-testing correction will be applied (e.g., Benjamini–Hochberg) where applicable. Data will be appropriately normalized and, if necessary, transformed. Dimension-reduction techniques, including principal component analysis (PCA), principal coordinates analysis (PCoA) or non-metric multidimensional scaling (NMDS), will be used to visualize sample clustering and compositional differences.

### 4.2 Monitoring

Because the trial objectives and a short follow-up period, monitoring responsibilities will rest with the study protocol team and the local Institutional Review Board (IRB); no independent Data Monitoring Committee (DMC) will be established. Study data will be accessible only to authorized and trained staff via unique, password-protected accounts; electronic data will be stored on secure servers with appropriate technical safeguards.

## 5 Discussion

The aging global population will likely produce a rising incidence of Parkinson’s disease (PD) with attendant social and economic burdens. Although pharmacological treatment has advanced, no durable disease-modifying therapy is currently available, underscoring the need to investigate accessible, cost-effective non-pharmacological strategies. The PRIME study addresses this gap by evaluating the synergistic effects of combined nutritional and physical-activity interventions on the GM and on both motor and non-motor clinical outcomes. By adopting a factorial design, the trial can disentangle the effects of each intervention alone and in combination, thereby providing robust comparative data. Clarifying the temporal and potentially causal relationships between gut microbiome alterations and PD — and how these relationships respond to diet and exercise — may facilitate the identification of an easily accessible, non-invasive stage specific biomarker for PD, a need that is unmet at present. The study’s findings are expected to inform future non-pharmacological protocols, including multidisciplinary rehabilitation programs, aimed at slowing disease progression, reducing symptom burden, and enabling earlier diagnosis. When implemented through well-structured rehabilitation pathways, these interventions are anticipated to improve patients’ independence and quality of life, and to result in fewer hospital admissions and lower long-term care costs. These outcomes will have immediate translational potential for clinical practice and meaningful benefits for health-care systems.

## Supplementary material

Supplementary Table 1 Details of activities and list of assessments across the study timepoints.

Supplementary Figure 1 Planned participant Flow-diagram of PRIME study, to be completed at final analysis.
