# Supplementary figures and images for: Effects of combined Mediterranean diet and physical activity intervention on the gut microbiome and disease progression in individuals with Parkinson’s disease: study protocol for a multicenter, randomized controlled pilot study (PRIME study)
Source: Front Aging Neurosci. 2026 Mar 25;18:1743490. doi: 10.3389/fnagi.2026.1743490 (PMC13057374; doi:10.3389/fnagi.2026.1743490)

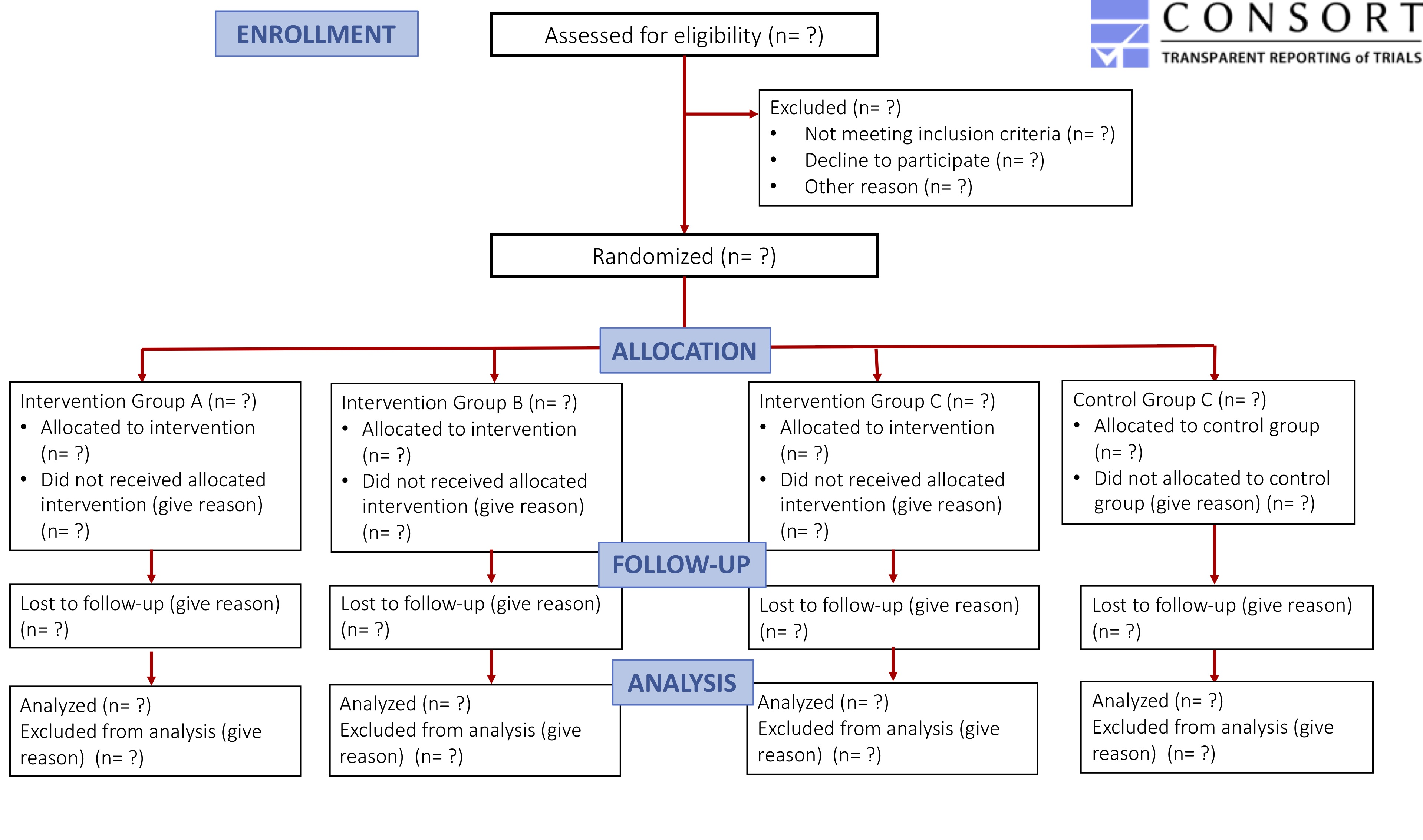

Supplement: Supplementary Figure 1 — Planned participant Flow-diagram of PRIME study, to be completed at final analysis. [file Image_1.png]
